# Understanding the pivotal roles of ACE2 in SARS-CoV-2 infection: from structure/function to therapeutic implication

**DOI:** 10.1186/s43042-022-00314-9

**Published:** 2022-06-19

**Authors:** Amir Pouremamali, Abouzar Babaei, Somayeh Shatizadeh Malekshahi, Ardeshir Abbasi, Nastaran Rafiee

**Affiliations:** 1 Department of Virology, Faculty of Medical Sciences, Tarbiat Modares University, Tehran, Iran; 2 Department of Immunology, Faculty of Medical Sciences, Tarbiat Modares University, Tehran, Iran

**Keywords:** SARS-CoV-2, Spike glycoprotein, Receptor-binding domain, Angiotensin-converting enzyme 2, Structure, Function, Inhibitor, Activator

## Abstract

In December 2019, a novel respiratory tract infection, from severe acute respiratory syndrome coronavirus 2 (SARS-CoV-2), was detected in China that rapidly spread around the world. This virus possesses spike (S) glycoproteins on the surface of mature virions, like other members of coronaviridae. The S glycoprotein is a crucial viral protein for binding, fusion, and entry into the target cells. Binding the receptor-binding domain (RBD) of S protein to angiotensin-converting enzyme 2 (ACE 2), a cell-surface receptor, mediates virus entry into cells; thus, understanding the basics of ACE2 and S protein, their interactions, and ACE2 targeting could be a potent priority for inhibition of virus infection. This review presents current knowledge of the SARS-CoV-2 basics and entry mechanism, structure and organ distribution of ACE2, and also its function in SARS-CoV-2 entry and pathogenesis. Furthermore, it highlights ACE2 targeting by recombinant ACE2 (rACE2), ACE2 activators, ACE inhibitor, and angiotensin II (Ang II) receptor blocker to control the SARS-CoV-2 infection.

## Background

In December 2019, a newly emerged coronavirus disease 2019 (COVID-19) appeared for the first time in Wuhan, China [1]. Since then, this devastating global health and economic challenge rapidly spread throughout the world and the world health organization (WHO) officially declared it as a global pandemic [2].Genome sequencing analysis of respiratory specimens identified that severe acute respiratory syndrome coronavirus 2 (SARS-CoV-2) is the cause active pathogen for COVID-19 [3]. According to the WHO statistics, with more than 500,000,000 confirmed cases of COVID-19 and over 6 million deaths by May 2022, SARS-CoV-2 is still the most challenging health issue, globally. Similar to other countries, so far, Iran has been involved in the pandemic by > 7,200,000 and 141,000, COVID-19 cases and deaths, respectively.

Coronaviruses (CoVs) belong to the Coronaviridae family with a single-stranded, positive-sense RNA (ssRNA) genome inside a large enveloped structure [4, 5]. This huge virus family is divided into four genera named Alpha, Beta, Gamma, and Delta [6, 7]. The coronavirus genus Beta includes highly pathogenic viruses such as severe acute respiratory syndrome coronavirus (SARS-CoV), Middle East respiratory syndrome coronavirus (MERS-CoV), and the novel SARS-CoV-2 and low pathogenic viruses including HCoV-OC43 and HCoV-HKU. Highly pathogenic viruses are commonly zoonotic in origin and cause lower respiratory tract infection while low pathogenic viruses are only endemic in humans and often lead to common colds [8, 9].

Furthermore, genome sequencing of SARS-CoV-2 revealed that this virus has a 29.9 kb genome size which shares around 80% and 96% identity with SARS-CoV and bat coronavirus at nucleic acid level, respectively, suggesting that SARS-CoV-2 has originated from bat SARS-like coronavirus [10, 11]. Although highly pathogenic betacoronaviruses share similarities with each other [12], the novel SARS-CoV-2 infection has a powerful human-to-human transmission capacity [10], which is highly infectious [13, 14], and also effectively evades from immune responses [15] compared to SARS-CoV or MERS-CoV. The clinical manifestations of COVID-19 ranges from mild and common symptoms including fever, dry cough, dyspnea, rhinitis, myalgia and/or fatigue and typically progresses to severe outcomes such as pneumonia, RNAaemia (SARS-CoV-2 RNA in serum), heart injury, acute respiratory distress syndrome (ARDS) (10–20% of patients), and death (0.1–15.4% of patients) [16–18].

The SARS-CoV-2 infection starts by binding of viral receptors to angiotensin-converting enzyme 2 (ACE2) in the surface of alveolar epithelial cells [19–21]. Meanwhile, it has been shown that binding affinity of SARS-CoV-2 to ACE2 is about 10–20 times higher than the interaction of SARS-CoV to this receptor [22]. In addition, this cellular receptor plays a very important role in the pathogenesis of new SARS-CoV-2 infection [23, 24].

Due to recombination of SARS-CoV-2 RNA sequences and involvement of host enzymes in viral RNA editing, the SARS-CoV-2 spike protein has undergone many mutations that led to emergence of different variants including alpha, beta, gamma, delta, omicron, etc., with increased reproduction number (R) and transmission potential [25–27].

From 95% of mRNA vaccines efficacy like Pfizer-BioNTech to approximately 60% or less effective vaccines such as Soberana 02 vaccine, all approved vaccines have demonstrated to be effective in reducing the number of mortality and morbidity. Since worldwide administration of different SARS-CoV-2 vaccine platforms, including mRNA, viral vector, inactivated virus, and live attenuated virus vaccines, the elevated neutralizing antibodies in vaccinated individuals raised hope for reducing the risk of infection; however, the appearance of delta and omicron variants has challenged vaccines efficacy [26, 28].

Also, there are no available specific medications with Food and Drug Administration (FDA) approvals against COVID-19, except some SARS-CoV-2-targeting monoclonal antibodies which is just authorized under an emergency use authorization (EUA) and are not approved by FDA. So, finding new protective vaccines and antiviral agents for curing this infection is one of the urgent mankind’s needs. In this review we addressed the role of ACE2 in SARS-CoV-2 infection and pathogenesis. In addition, the efficiency of ACE2 analogs for the treatment of COVID-19 is explained in the present context.

## SARS-CoV-2 and cellular receptors

**Fig. 1:**
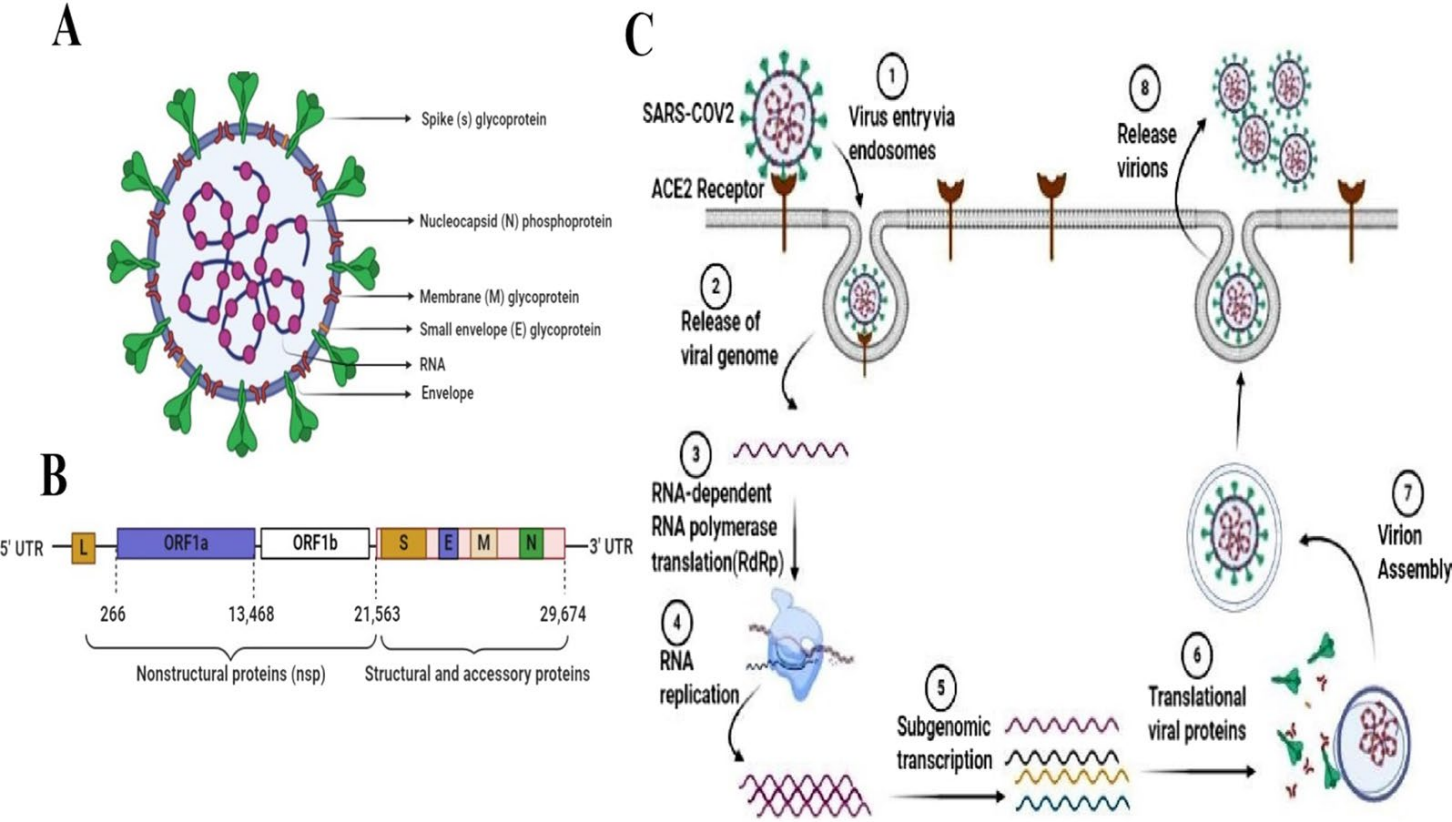
Schematic representation of SARS-COV-2 structure and life cycle. **A** structure of SARS-CoV-2; the crown-like spike proteins (S) are placed on the outside of the viral envelope. **B** SARS-CoV-2 genomic structure; SARS-CoV-2 genome consists of the 5′-untranslated region (5′-UTR), nonstructural proteins region, structural and accessory proteins region, and the 3′-untranslated region (3′-UTR). **C** life cycle of SARS-CoV-2 in host cells; virus starts its life cycle by binding of S1 protein to the cellular receptor ACE2 and then proteolytic cleavage in the S protein facilitates the fusion of viral and cellular membranes by S2 protein. After the release of the viral genome into the cytoplasm, viral RNA replicates, and then viral proteins translate from the RNA and then viral proteins and genome are assembled into virions in the endoplasmic reticulum and Golgi. Eventually, Virions are transported by vesicles to near the cell membrane and released out of the infected cell by budding from the cytoplasmic membrane

Coronavirus is named for the crown-like spike proteins (S) outside of the viral envelope (Fig. 1A) [29, 30]. Genome analysis studies of CoVs indicated that structural proteins are encoded by the spike (S), envelope, membrane, and nucleocapsid genes (Fig. 1B) [31]. The S protein [1273 amino acids], presented on the surface of mature CoVs in homotrimer form, is the main viral mediator in virus entry into host cells [32]. This viral protein consists of two functional subunits: (i) the S1 subunit, containing a receptor-binding domain (RBD) which mediates virus binding to host cells, and (ii) the S2 subunit that fuses membranes of virus and host cell [33, 34].

It has been shown that the SARS-CoV-2 S protein shares around 80% identity in amino acid level with the SARS-CoVs protein and these two viruses also show nearly similar 3D structure [35]. However, some reports confirmed that little but functionally important differences at residues 331–524 of SARS-CoV-2-RBD, enable this virus to bind to cellular receptors with higher affinity than SARS-CoV [8, 36–38]. The transmembrane protease serine 2 (TMPRSS2) cleaves S protein at the S2´ site, located between S1 and S2. This cleavage leads to extensive structural changes in S2 protein and actives it to fuse with the membrane and completes SARS-CoV-2 internalization through endocytosis process (Fig. 1C). All these data indicate that viral S protein and human ACE2 play important roles in establishing SARS-CoV-2 infection and are required for virus entry [10, 39–41]. Furthermore, an in vivo study confirmed that the knockout of the ACE2 gene could inhibit SARS-CoV-2 infection in murine epithelial cells [40]. Thus, blocking of virus attachment to ACE2 receptors can be considered as a therapeutic option.

## ACE2 structure and function

**Fig. 2:**
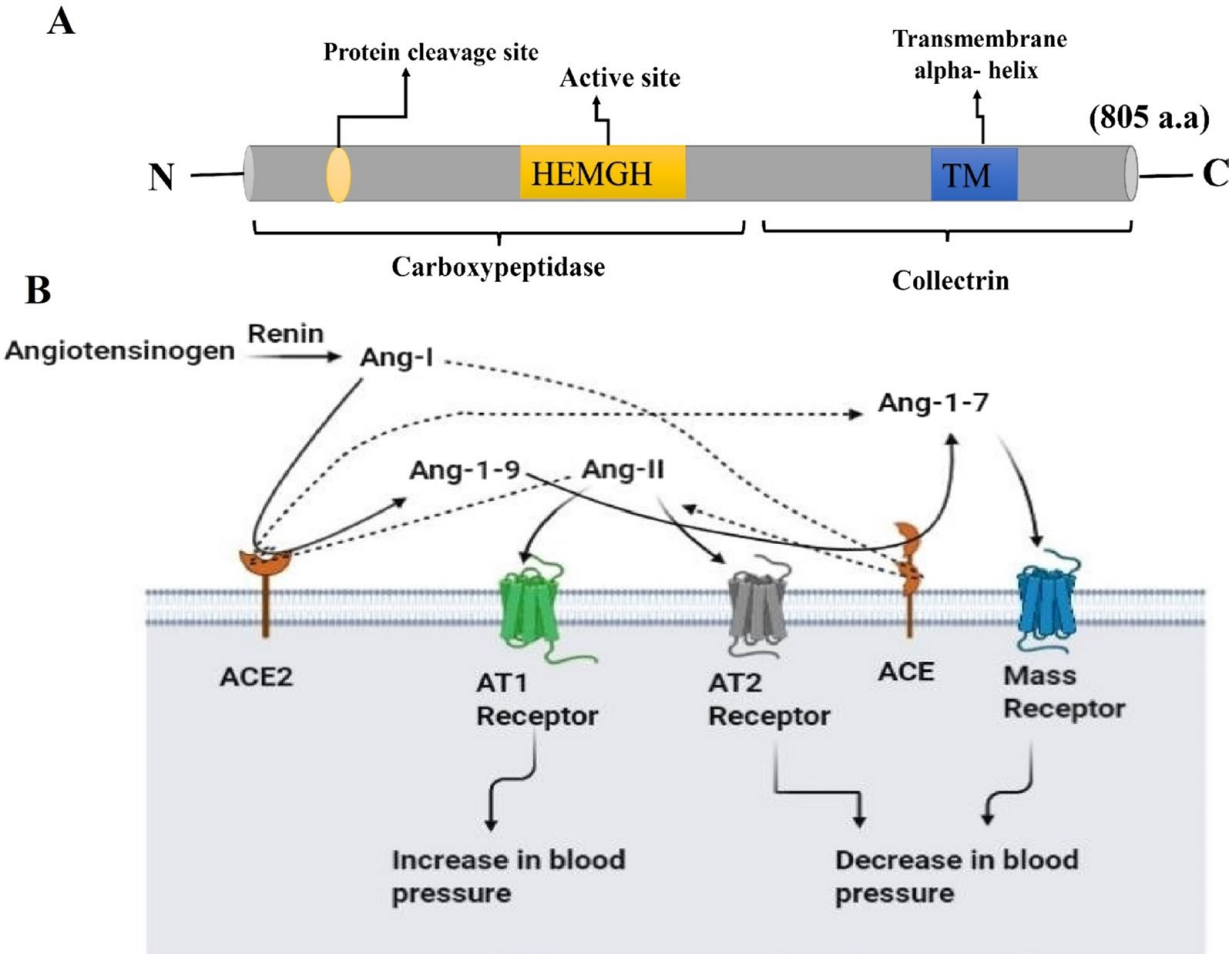
ACE2 structure and its function in the renin–angiotensin–aldosterone system (RAAS). **A** ACE2 structure; N-terminal domain includes single peptide, protein cleavage site, and active site and C-terminal domain contain transmembrane alpha-helix. **B** Schematic overview of ACE2 functions in the renin–angiotensin–aldosterone system (RASS)

In 2000, scientists discovered a new homolog of ACE and named it ACE2. This homolog is encoded by ACE2 gene, placed in chromosome Xp22, and contains 18 exons [42]. ACE2 as a cell-surface receptor, like ACE, is a transmembrane protein that consists of two domains: (i) N-terminal contains a catalytic site, and (ii) C-terminal possesses a transmembrane anchor (Fig. 2A). Structurally, ACE has 2 active catalytic sites while ACE2 possesses only a single active site in the N-terminal domain, and also there is no similarity between the C-terminal domain of ACE2 with ACE [43, 44]. In ACE2, the protease domain (PD) in the N-terminal portion performs all peptidase activities and the C-terminal domain, as a strong anchor, connects the C-terminal domain to the cell membrane and regulates the amino acid transporters trafficking [45].

Functionally, ACE2 and ACE are carboxypeptidases that play axial roles in renin–angiotensin–aldosterone system (RAAS) to regulate hemostasis in the human body (Fig. 2B) [46]. ACE2 has only one single catalytic site that acts as a simple carboxypeptidase while its ACE isoform contains two active domains that cleave amino acids, located in the carboxyl end of peptides [47]. However, these structural and functional differences are important in their enzymatic activities. In RAAS, angiotensinogen, a precursor peptide secreted from the liver, is cleaved by renin and converted to angiotensin I (Ang I) decapeptide, then ACE cleaves 2 amino acids from Ang I to produce an angiotensin II (Ang II) octapeptide, while ACE2 converts Ang I to angiotensin (1–9) [Ang (1–9)] by cleaving one amino acid of that. ACE catabolizes Ang (1–9) into Ang (1–7). ACE2 can also cleave Ang II to form Ang (1–7). Ang (1–7) binds to Mas receptors and performs its activity (Fig. 2B) [48, 49].

ACE2, as a negative regulator of the RAS, has vasodilatory, anti-inflammatory, and anti-fibrotic impacts and counter-balances the ACE/AngII/angiotensin receptor 1 (AT1 R) pathway [50, 51]. Apart from ACE2 systemic actions, this enzyme has regulatory effects in the heart, kidney, lung, and gastrointestinal tracts. In this way, ACE2 controls the metabolism of bradykinin in the lungs, orchestrates the amino acid absorption in the kidney and also modulates the insulin secretion from pancreatic cells. This enzyme regulates the homeostasis of amino acids, the expression of peptides, and local innate immune responses in the gut [52]. Furthermore, studying the newly emerged SARS-CoV-2 infection has identified the ACE2 as a crucial receptor for virus entry [53].

## Organ distribution of ACE2

ACE2 proteins are active and extensively expressed in a wide range of organs and tissues of the human body. In the respiratory tract, ACE2 widely presents in the epithelium of basal and oral mucosa, nasopharynx, and alveolar epithelial cells of lungs and bronchial and also exerts protective effects on lung [54]. ACE2 prevents bradykinin from binding to its receptor, prohibits releasing pro-inflammatory cytokines, lung injury, and inflammation [55]. Also, it has been shown that acute lung injury and inflammatory lesions in the respiratory tract markedly increased in the ACE2 knockout mice and these implications disappeared after injection of recombinant ACE2 [56]. Furthermore, different organs of the gastrointestinal tract including, the stomach, colon, duodenum, jejunum, and ileum are strongly capable to express ACE2. Besides, the ACE2 localization in the endothelial cells and smooth muscle cells of cardiovascular tissue, the kidney, skin, male testis, and female breast are highly positive for this aminopeptidase [57–59]. In contrast, ACE2 is not detectable in lymphoid tissues and hepatobiliary structures including the spleen, thymus, lymph nodes, bone marrow, and even immune system cells [60].

## ACE2 and SARS-CoV-2 pathogenesis

Respiratory tract cells are the first target for starting SARS-CoV-2 infection because of high expression level of ACE2 [61]. Following infection, virions infect epithelial cells, complete their life cycle, and produce progenies by destroying the infected cells which results in limited lung injury. Then, the local and innate immune cells including dendritic cells and macrophages which serve as sentinel cells present the SARS-CoV-2-infected destroyed cells to adaptive cells [62]. Asymptomatic or mild stages of infection are seen in around 80% of infected individuals [63, 64]. Unfortunately, in 20% of patients, the disease progresses and causes severe respiratory complications including ground-glass opacities, RNAaemia, and ARDS [18, 64, 65]. Severe respiratory symptoms are usually seen in patients who are elderly and have coexisted chronic diseases with weak immune responses and poor respiratory tract function [17, 52]. The cytokine storm resulted from the increased level of pro-inflammatory cytokines such as interleukins (IL-1, IL-6), interferon, and tumor necrosis factor (TNF-α), as immune response to virus infection, causes severe symptoms in the lung [66–68].

**Fig. 3:**
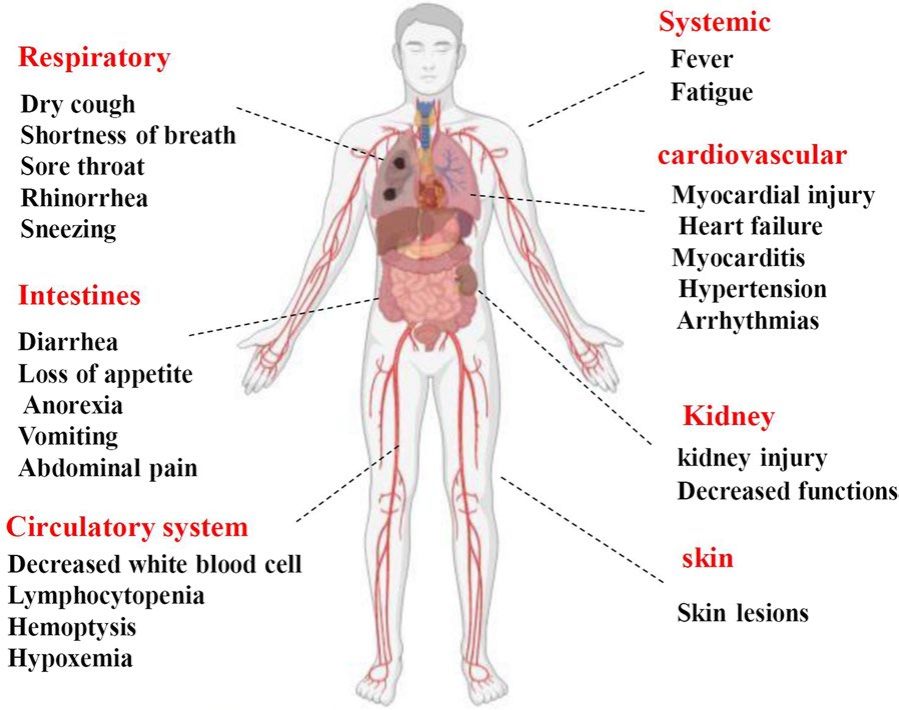
The systemic and respiratory manifestations of SARS-COV-2 infection

Although SARS-CoV-2 is known as a respiratory tract infectious agent, ACE2 distribution in the above-mentioned organs introduces it as a multi-organ pathogen with systemic and respiratory manifestations (Fig. 3) [61]. It has been shown that the presence of ACE2 on the surface of gut cells makes them susceptible to SARS-CoV-2 infection and causes symptoms including loss of appetite, anorexia, diarrhea, vomiting, and abdominal pain [69, 70]. In the cardiovascular system, ACE2 as a part of the RAAS system has a protective role, but SARS-CoV-2, when binds to this cellular receptor, may facilitate targeting of myocardial cells for the viral infection [62]. The reported SARS-CoV-2-associated cardiovascular outcomes include myocardial injury, heart failure, myocarditis, hypertension, diabetes, and arrhythmias [16, 71–73]. The mild-to-moderate proteinuria is the most common kidney complication in COVID-19 patients while acute kidney injury (AKI) is detectable in the severe form of SARS-CoV-2 infection [74]. Also, SARS-CoV-2 can cause variable skin lesions in > 20% of patients [75, 76]. However, it is not well known that these skin abnormalities may be primarily caused by viral invasion or secondary by induced immune responses and treatments [62]. Also, neurological [77], ocular [78, 79], hematological manifestations [80, 81], and endocrine abnormalities [82] were seen in the COVID-19 patients. Although these suggest that ACE2 may play critical roles in SARS-CoV-2 infection, new and more data are essentially needed for a precise understanding of SARS-CoV-2 pulmonary and extra pulmonary outcomes.

## ACE2 and respiratory infections

The COVID-19 symptoms range from an asymptomatic state to mild upper respiratory tract infection and severe pneumonia, ARDS, or even multi organ failure. ARDS is a syndrome characterized by the acute onset of hypoxemia ([PaO2/FiO2] B200 mmHg), with bilateral infiltrates on chest imaging, but without sign of left atrial hypertension [83]. The common risk factors associated with ARDS include pneumonia (virus, bacteria, fungus), aspiration of gastric contents, inhalational injury and sepsis, and major trauma that is classified into direct and indirect lung injury categories [83]. The treatment is mainly based on lung-protective ventilatory strategy and mortality has been markedly reduced with supportive treatment. However, there is no suggested pharmacological treatment [84]. An observational study in Wuhan, China, showed that 67–85% of COVID-19 patients were admitted to the intensive care unit (ICU), indicated severe ARDS, and the mortality rate in patients with ARDS was up to 61.5% [85]. Additionally, the clinical outcomes in patients with COVID-19 correlated with the severity of ARDS, and the mortality of patients with moderate and severe ARDS was higher than mild ARDS. Furthermore, there is increasing evidence that chronic obstructive pulmonary disease (COPD) may be a risk factor for more severe COVID-19 disease. Seven studies showed that COPD patients were also at higher risk of severe COVID-19 compared to patients without COPD (33.4%) [86].

ACE2 plays a significant role in the progression of ARDS. The roles of ACE and ACE2 have been investigated in an animal model of ARDS. They have shown that ACE activity was enhanced in ARDS, whereas ACE2 activity was reduced. This was correlated with enhanced levels of Ang II and reduced levels of Ang-(1–7). Hence, treatment with cAng-(1–7) decreased the severity of lung damage and improved lung function [87]. However, in mice models, ACE2 has been shown to protect animals from severe lung damage, caused by aspiration and sepsis [56]. Based on studies, ACE2 expression in the human airway epithelium was remarkably elevated in COPD patients. Interestingly, smoking status was meaningfully associated with ACE2 gene expression levels in the airway epithelium of COPD patients; for example, current smokers significantly had a higher gene expression than former and never smokers [88].

While the overexpression of the ACE2 may have a protective effect against acute lung injury, the upregulation of ACE2 might predispose patients to an increased risk for infection with the coronavirus, which uses this receptor for entry into target cells. This may partially explain the severity of COVID-19 disease in COPD populations [20]. It is worth noting that the upregulation of ACE2 alone does not support increased susceptibility or severity of the disease. Moreover, relatively low ACE2 expression levels in the bronchial epithelium are associated with more severe lung injury [88, 89].

## ACE2 regulation

Evidence suggests that the expression of the ACE2 is regulated by various pathways. Enhanced ACE2 expression can be protective in patients with diabetes, cancer, and cardiovascular disease [90]. Notably, ACE2 protein expression is significantly upregulated under stress conditions, including hypoxic conditions, treatment with IL-1β, and treatment with 5-amino-4-imidazole carboxamideriboside (AICAR). The analysis demonstrated that AICAR and IL-1β treatment upregulates and downregulates ACE2 expression, respectively [90]. The finding showed that cardiac ACE2 is increased following treatment by AT1 receptor blockers (ARBs), nevertheless, Endothelin-1 (ET-1) significantly reduce myocytes ACE2 mRNA levels to downregulate ACE2 activity [91].

ANG II considerably reduces ACE2 activity and downregulates mRNA levels of ACE2 in cardiac myocytes [92]. Another study showed that inhibition of Ang II synthesis altered ACE2 expression in mRNA levels [93]. Evidence suggests that there is a link between Ang II levels and the expression of ACE2 [94]. For example, under hypoxia conditions ACE2 transcription is reduced, on the other hand, hypoxia-induced HIF-1α upregulates ACE expression which in turn leads to higher concentrations of Ang II. Therefore, Ang II mediates the reduction in ACE2 mRNA and its activity [95]. Furthermore, the in vivo experiments have shown that Ang-(1–7) has antagonistic effects on ACE2 expression. In rat models, cardiac and renal ACE2 were decreased in response to Ang-(1–7) infusion [96]. Also, it has been shown that the administration of aldosterone or endothelin-1 significantly reduced myocyte ACE2 mRNA levels [91]. The effects of all trans-retinoic acid on gene and protein expression of ACE2 have been investigated in rats, and a significant upregulation of ACE2 expression in mRNA and protein levels was observed in the heart and kidney [97]. Plasma levels of circulating ACE2 have been reported to be very low or even undetectable in healthy individuals; however, ACE2 levels were significantly increased in the presence of cardiovascular disease. In two distinct cohorts of heart failure patients with COVID-19, men had higher plasma concentrations of ACE2 than women, hence ACE inhibitors and angiotensin receptor blockers were associated with lower plasma concentrations of ACE2, but mineralocorticoid receptor antagonists were associated with higher concentrations [98].

The sex difference is an important risk factor among COVID-19 patients, compared to women, men, infected with the SARS-CoV-2, experience more severe disease and higher mortality rates [99]. This difference could be derived from increased ACE2 expression in male that plasma levels of ACE2 were higher in males than in females and also this difference could be related to a genetic polymorphism in ACE2 [100]. Previous studies reported that the expression of ACE2 depends on age and sex. As observed in rat lungs, ACE2-expression in females and younger animals is higher than in males and adults. Therefore, it is predictable that higher levels of ACE2 are present in the lungs of children and young people than in adults and old people. Therefore, it can be concluded that there is a direct relationship between the overexpression of ACE2 in children, young people, and women with the lower pathology and morbidity rate of COVID 19. The severity of COVID 19 symptoms can be related to ACE2 genetic polymorphisms that are reported in different populations worldwide [101, 102].

Other studies have shown that aldosterone and estrogens can regulate the expression of ACE2 in cell lines and animal models. In a mouse model of SARS-CoV infection, female mice had lower viral titers and less severe disease and cytokine production; the endogenous estradiol was an important factor in this protection [103]. 17β-estradiol-treated cells expressed lower levels of ACE2 mRNA compared to controls. They showed that sex hormones play critical roles in the regulation of cellular components required for SARS-CoV-2 infectivity and the ability to cause life-threatening disorders [104].

## ACE2 targeting for treatment

### Recombinant ACE2 (rACE2)

It has been suggested that inhibiting the interaction of SARS-CoV-2 with ACE2 might be used as a therapeutic method in the treatment of patients with COVID-19 [105]. The use of human recombinant soluble ACE2 (hrsACE2) to neutralize the virus and prevent lung damage is now under clinical investigation [106].

**Fig. 4:**
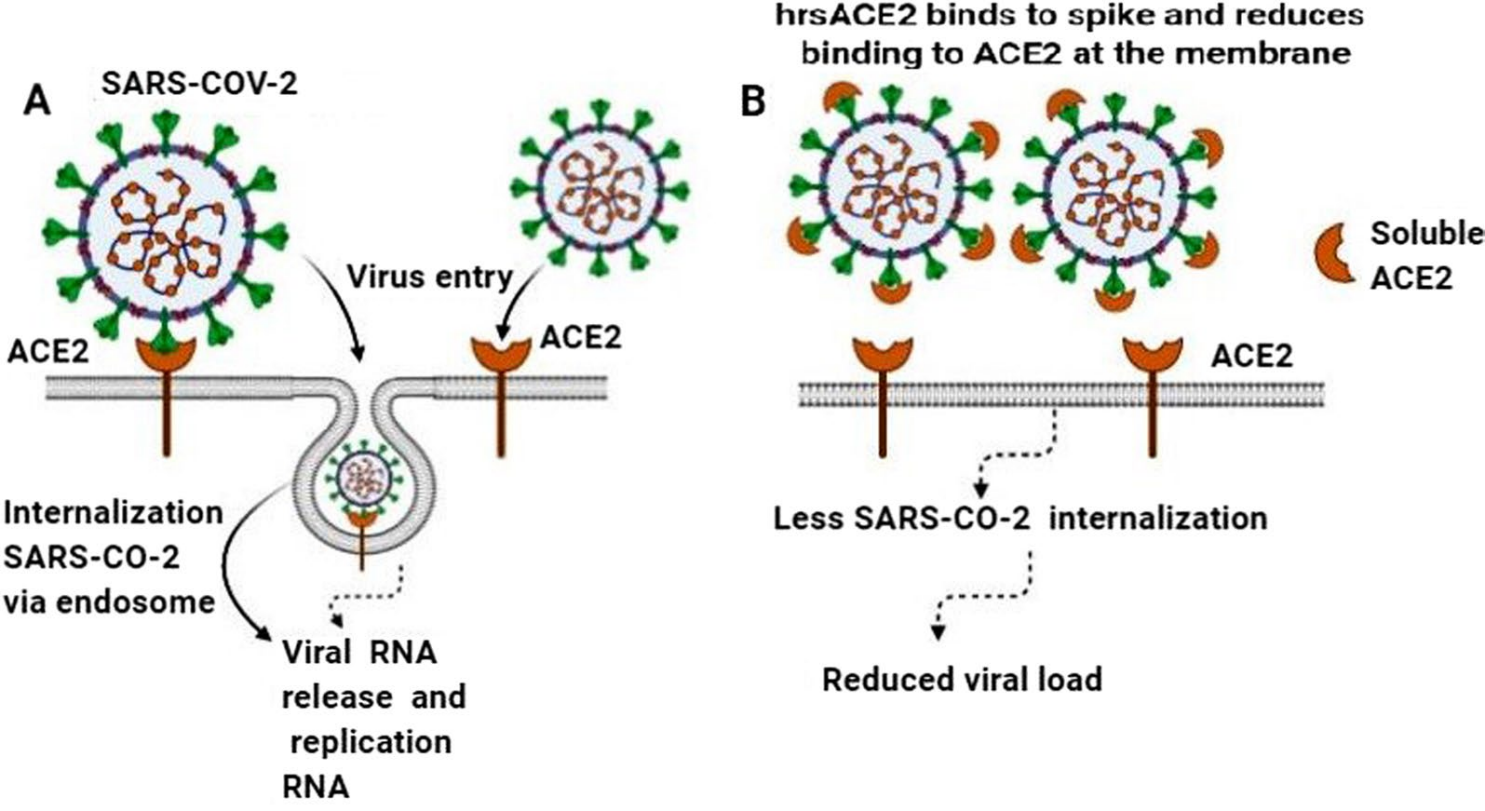
hrsACE2 inhibits SARS COV-2 replication. **A** SARS-CoV-2 by spike protein directly binds to the ACE2 receptor and then inter to the host cell by endocytosis. **B** The covering of SARS-CoV-2 spike protein by hrsACE2 lead to the prevention of the interaction between SARS-CoV-2 and ACE2 and subsequently prevents SARS COV-2 replication in the host cell

Studies have demonstrated that the early stages of SARS-CoV-2 infection can be inhibited by hrsACE2. These data demonstrated that hrsACE2 inhibits SARS-CoV-2 attachment to the host cells. Soluble ACE2 binds to spike protein and reduces binding to ACE2 on the cell membrane and prevents SARS-COV-2 replication (Fig. 4). This inhibitory action of hrsACE2 is dependent on the amount of virus, present in the initial inoculums, and the dose of hrsACE2 [105].

### ACE2 activators

Decrease in pulmonary ACE2 activity is associated with pneumonia; in contrast, its activation can inhibit hyperoxia-induced lung injury by inhibiting the inflammatory response and oxidative stress [107]. Since upregulation of ACE2 has a protective role against severe lung injury, including ARDS and COPD, the development of an ACE2 activator could be a potential therapeutic strategy against COVID-19.

Xanthenone (XNT) or diminazeneaceturate (DIZE) and resorcinolnaphthalein have the ability to activate ACE2 [102]. XNT or DIZE is an anti-parasitic drug for treating trypanosomiasis. In macrophages, DIZE downregulates the MAPK/ERK and STAT phosphorylation (signaling molecules), resulting in the downregulation of IL-6, IL-12, and tumor necrosis factor-α (TNF-α) which ultimately reduces inflammatory responses. Treatment with DIZE reduces the expression of CD25 (T cell marker) in spleens of Trypanosoma infected mice, suggesting that DIZE dampens immune system activation [108]. According to experiments, glucocorticoids such as hydrocortisone, prednisolone, methylprednisolone, and dexamethasone showed the strongest effect on activating ACE2 and inhibiting IL-6 [109].

### ACE inhibitor and Ang II receptor blocker

High blood pressure in patients with COVID -2019 is one of the symptoms that is associated with worse clinical outcomes. ACE inhibitor (ACEI) and angiotensin II receptor blocker (ARB) are widely prescribed for treating high blood pressure [110]. Since commonly used antihypertensive medications may upregulate ACE2 receptors, these concerns have been raised that their use may result in increased morbidity and mortality rate. Lei Fang et al. suggested that patients with cardiac diseases, hypertension, and/or diabetes, who were treated with ACE2 activating drugs, would be at risk for severe COVID-19 infection, because treatment with ACEIs and ARBs may upregulate ACE2 expression [53]. On the contrary, several clinical studies have reported that ACEi/ARB used in COVID-19 patients with hypertension does not worsen COVID-19 disease severity or mortality. Data from clinical studies suggest that ACEI/ARB administration does not increase ACE2 expression and the risk of COVID-19 complications [111, 112]. Other compounds that may increase the expression of the ACE2 receptor include Vitamin C, metformin, resveratrol, vitamin B3, and vitamin D [113].

## Conclusion

We summarized the role of ACE2 in the pathogenesis, progression, and treatment of COVID-19 infection. ACE2 controls the metabolism of bradykinin, regulates amino acid transportation, and also modulates insulin secretion. This enzyme is the receptor for the SARS-CoV-2 and has a local protective effect on tissue injury and is implicated in the progression and prognosis of COVID-19. Sex difference is an important risk factor, compared to women; men have more severe disease and higher mortality rates. This difference may be derived from increased ACE2 plasma levels expression in men than in women, while higher tissue expression of this receptor seems to be beneficial for preventing exacerbated inflammatory response during infection. As well as, based on findings, the severity of COVID 19 symptoms can be related to ACE2 genetic polymorphisms in different populations.

Although the global vaccination program seems to be effective in controlling hospitalization rate and deaths, caused by SARS-CoV-2, it is unable to prevent the development of new variants like delta or omicron, which have shown more infectivity or transmissibility. As new variants have relatively reduced vaccine’s potency in increasing neutralizing antibodies, the requisite of ACE2 inhibitory drugs, discussed in this review, becomes more of interest and can be much more effective in curing patients than those which do not target ACE2. Prescribing drugs that may affect ACE2, such as ACEI, ARB, or ACE2 activators and targeting ACE2 by using hrsACE2 can be a potential therapeutic strategy for COVID-19; however, more clinical studies are needed to confirm the effectiveness of these methods.

## Data Availability

Not applicable.

## Abbreviations

ACE: Angiotensin-converting enzyme
ACE 2: Angiotensin-converting enzyme 2
ACEI: Ace inhibitor
AICAR: 5-Amino-4-imidazole carboxamideriboside
AKI: Acute kidney injury
ANG I: Angiotensin I
ANG II: Angiotensin II
ARB: Angiotensin II receptor blocker
ARBS: At1 receptor blockers
ARDS: Acute respiratory distress syndrome
AT1 R: ACE/AngII/angiotensin receptor 1
AT1 R: Theace/AngII/angiotensin receptor 1
COPD: Chronic obstructive pulmonary disease
COVID-19: Coronavirus disease 2019
COVS: Coronaviruses
DIZE: Diminazeneaceturate
ET-1: Endothelin-1
FDA: Food and drug administration
HRSACE2: Human recombinant soluble ACE2
ICU: Intensive care unit
IL: Interleukins
MERS-COV: Middle East respiratory syndrome coronavirus
PD: Protease domain
RAAS: Renin-angiotensin-aldosterone system
RACE2: Recombinant ACE2
RBD: Receptor-binding domain
SARS-COV: Severe acute respiratory syndrome coronavirus
SARS-COV-2: Severe acute respiratory syndrome coronavirus 2
TMPRSS2: Transmembrane protease serine 2
TNF-α: Tumor necrosis factor
WHO: World Health Organization
XNT: Xanthenone
